# Notch signaling in astrocytes mediates their morphological response to an inflammatory challenge

**DOI:** 10.1038/s41420-019-0166-6

**Published:** 2019-04-03

**Authors:** Estefania Acaz-Fonseca, Ana Ortiz-Rodriguez, Iñigo Azcoitia, Luis M. Garcia-Segura, Maria-Angeles Arevalo

**Affiliations:** 1 Instituto Cajal, Consejo Superior de Investigaciones Científicas (CSIC), Madrid, Spain; 2 CIBER de Investigación Biomédica en Red de Fragilidad y Envejecimiento Saludable (CIBERFES), Instituto de Salud Carlos III, Madrid, Spain; 3 Department of Cell Biology, Faculty of Biology, Universidad Complutense, Madrid, 28040 Spain

## Abstract

In the nervous system, Notch pathway has a prominent role in the control of neuronal morphology and in the determination of the astrocyte fate. However, the role of Notch in morphological astrocyte plasticity is unknown. Here, we have explored the role of Notch activity on the morphological reactivity of primary astrocytes in response to LPS, an inflammatory stimulus. We found that LPS induces reactive astrocyte morphology by the inhibition of Notch signaling via NFκB activation and Jagged upregulation. In contrast, IGF-1, an anti-inflammatory molecule, inhibits LPS-induced reactive astrocyte morphological phenotype by enhancing Notch signaling through the inhibition of NFκB and the activation of MAPK. Therefore, Notch signaling pathway emerges as a mediator of the regulation of astrocyte morphology by inflammatory and anti-inflammatory stimuli.

## Introduction

Reactive astrogliosis involves several processes that astrocytes undergo under pathological conditions^1–4^. The alterations suffered by reactive astrocytes vary with the nature and severity of the insult. Modest metabolic stimulus, infections, inflammatory processes or mild trauma induce moderate reactive astrogliosis characterized by changes in the molecular expression of pro-inflammatory cytokines together with cellular hypertrophy. However, in severe central nervous system (CNS) injury models, astrogliosis also involves cell proliferation and scar formation^3^.

Astrocytic hypertrophy of reactive astrocytes is characterized by an increase in the number, thickness, and length of the main cellular processes, which also present a greater content in GFAP bundles than in nonreactive astrocytes^5^. Although hypertrophy of astrocytes has been profusely studied, the signaling mechanisms that regulate morphological aspects of reactive astrogliosis remain unclear.

Notch1 receptor and ligands, Delta-like-1 (Dll-1) and Jaggeg-1 (Jag-1), have been extensively studied in relation with cell fate specification of neurons^6–8^, vascular smooth muscle cells^9^, pancreatic endocrine cells^10^, and astrocytes^11,12^. In addition, Notch signaling regulates the expression of molecules involved in the regulation of cell morphology in developing neurons^13,14^. The canonical trans-activation of the Notch pathway starts with the binding of the extracellular domain of the ligand to the extracellular domain of the receptor expressed in an adjacent cell. This allows a conformational change in the receptor that favors its cleavage by metalloproteases and by the enzymatic complex γ-secretase, resulting in the release of the intracellular domain of Notch (NICD), the active fragment of the receptor. NICD is then translocated into the cell nucleus, where it initiates the transcription of Notch target genes, mainly hairy and enhancer of split (HES)-1 and 5^6,15^, the main effectors of the pathway in the CNS^16^. Hes-1 and Hes-5 play a crucial role in neurogenesis, gliogenesis, neuritogenesis as well as in the development of sensory organs^17–19^. In the adult brain, Notch is involved in long-term memory^20^, dendritic plasticity^8^, synaptic plasticity^21^, and postnatal neurogenesis^22^.

Even if Notch functions in differentiated glial cells have not been deeply investigated, it is clear that it plays a role in neuroinflammation. For instance, Notch regulates microglia activation and pro-inflammatory cytokine release by its interaction with NFκB^23,24^. It has also been shown that hypertrophic astrocytes express Jag-1 in vivo^25^ and that the intermediate filaments GFAP and vimentin control Notch pathway activity in astrocytes^5^. Furthermore, Notch pathway regulates proliferation in reactive astrocytes surrounding an ischemic lesion^26,27^. However, the implication of Notch signaling in the morphological changes experimented by reactive astrocytes has not been explored previously.

In the present study, we have assessed whether Notch signaling is involved in the activation of astrocytes by an inflammatory challenge: the treatment with the bacterial endotoxin lipopolysaccharide (LPS). We have also explored whether Notch signaling in astrocytes is regulated by insulin-like growth factor 1 (IGF-1), since this factor reduces the astrocytic response to inflammatory stimuli and their expression of inflammatory mediators such as interleukin 6 (IL-6), tumor necrosis factor-α (TNF-α), interleukin-1β (IL-1β), toll-like receptor 4, and iNOS^28,29^.

## Results

### LPS induces a reactive inflammatory phenotype in primary astrocytes

**Fig. 1:**
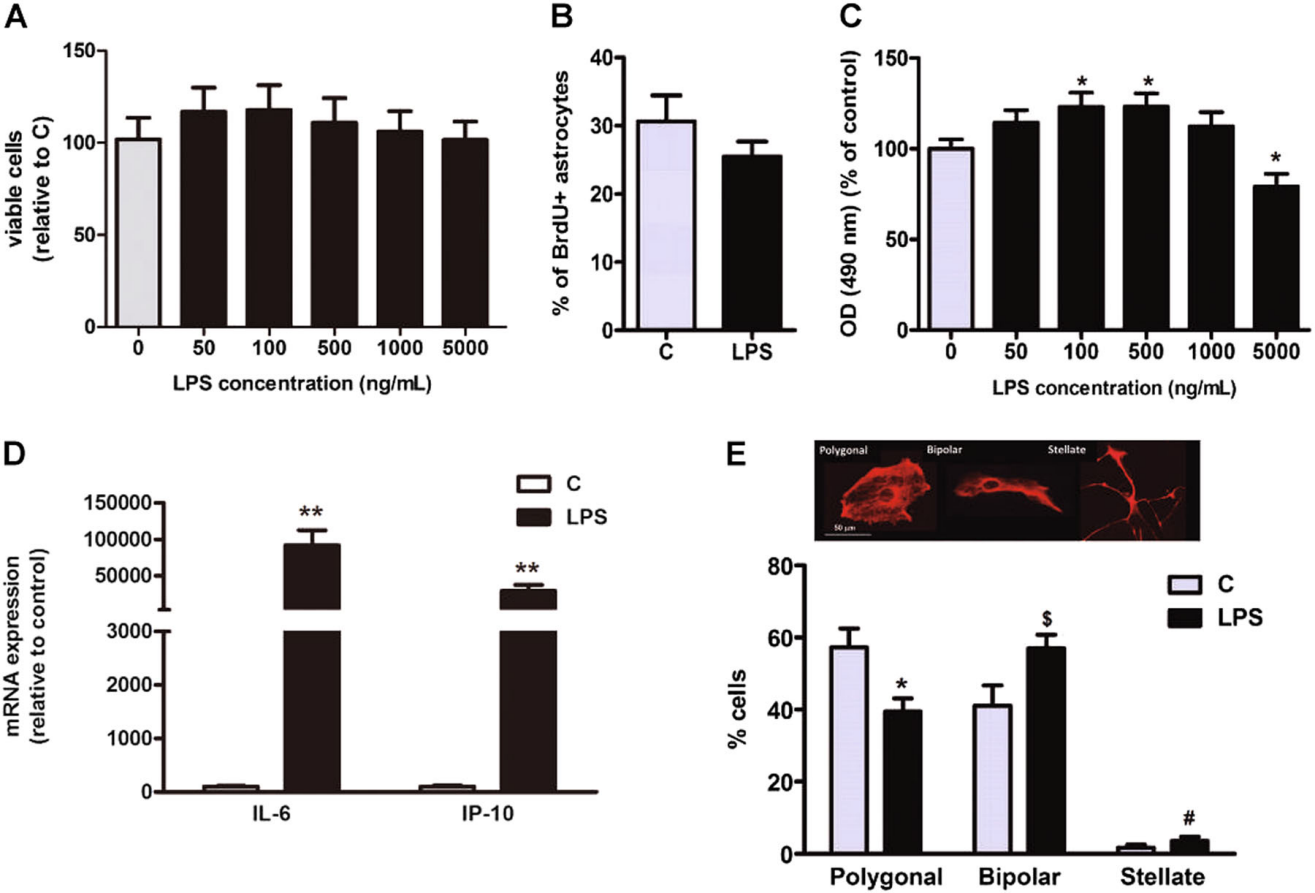
LPS stimulation of primary astrocytes. **a** LPS stimulation (500 ng/mL) during 24 h preserves cell viability as it was evaluated by FDA fluorescence emission and **b** it does not modify cell proliferation analyzed by immunocytochemistry against BrdU. **c** MTS oxidation rate (mitochondrial respiration) is modified by LPS. * Significant differences (*p* < 0.05) versus control (Student’s *t*-test, *n* = 4). **d** LPS treatment induces the transcription of pro-inflammatory cytokines; ** significant differences (*p* < 0.01) versus control (Student’s *t-*test, n = 4). **e** Astrocytes exposed to LPS display a significant change in their morphology, transitioning from a polygonal shape towards more elongated (bipolar and stellate) shapes. Immunofluorescence images show an example of the three types of astrocytes morphology in cultures, after immunostaining with an antibody against GFAP. Graphs represent the percentage of each cell type in the culture. *significant differences (*p* < 0.05) versus control polygonal cells. ^$^significant differences (*p* < 0.05) versus control bipolar cells. ^#^significant differences (*p* < 0.05) versus control stellate cells (Student’s *t*-test, *n* = 4)

Reactive astrogliosis is a set of changes that occur in astrocytes in response to CNS injury or disease. We evaluated the use of mouse primary astrocyte cultures exposed to LPS during 24 h, as a model of in vitro astrogliosis^29^. The addition of LPS in concentrations ranging from 50 to 5000 ng/mL did not decrease cell viability, as assessed by a FDA test (Fig. 1a). In addition, immunocytochemistry of astrocytes treated with BrdU showed that LPS treatment did not change cell proliferation (Fig. 1b). However, 100 and 500 ng/mL LPS induced a significant increase in the optical density in the MTS test, while the dose of 5000 ng/mL LPS decreased the optical density compared to the control group (Fig. 1c). Based on the results of FDA test and of BrdU quantification, the changes observed in MTS test may represent differences in cell metabolic activity induced by LPS treatment.

Stimulation of astrocytes with 500 ng/ml LPS also enhanced the expression of two of the main pro-inflammatory factors released by reactive astrocytes: the cytokine IL-6 and the chemokine IP-10 (Fig. 1d). Moreover, this LPS dose induced changes in astrocyte morphology that are compatible with an increased reactivity. Thus, as shown in Fig. 1e, the percentage of cells with bipolar and stellate shapes was higher in the cultures treated with LPS than in the control conditions. Therefore, we established a model that at least mimics some of the main components of reactive astrogliosis by exposing primary cultures of cortical astrocytes to 500 ng/mL LPS for 24 h.

### Notch signaling is regulated by LPS in astrocytes

**Fig. 2:**
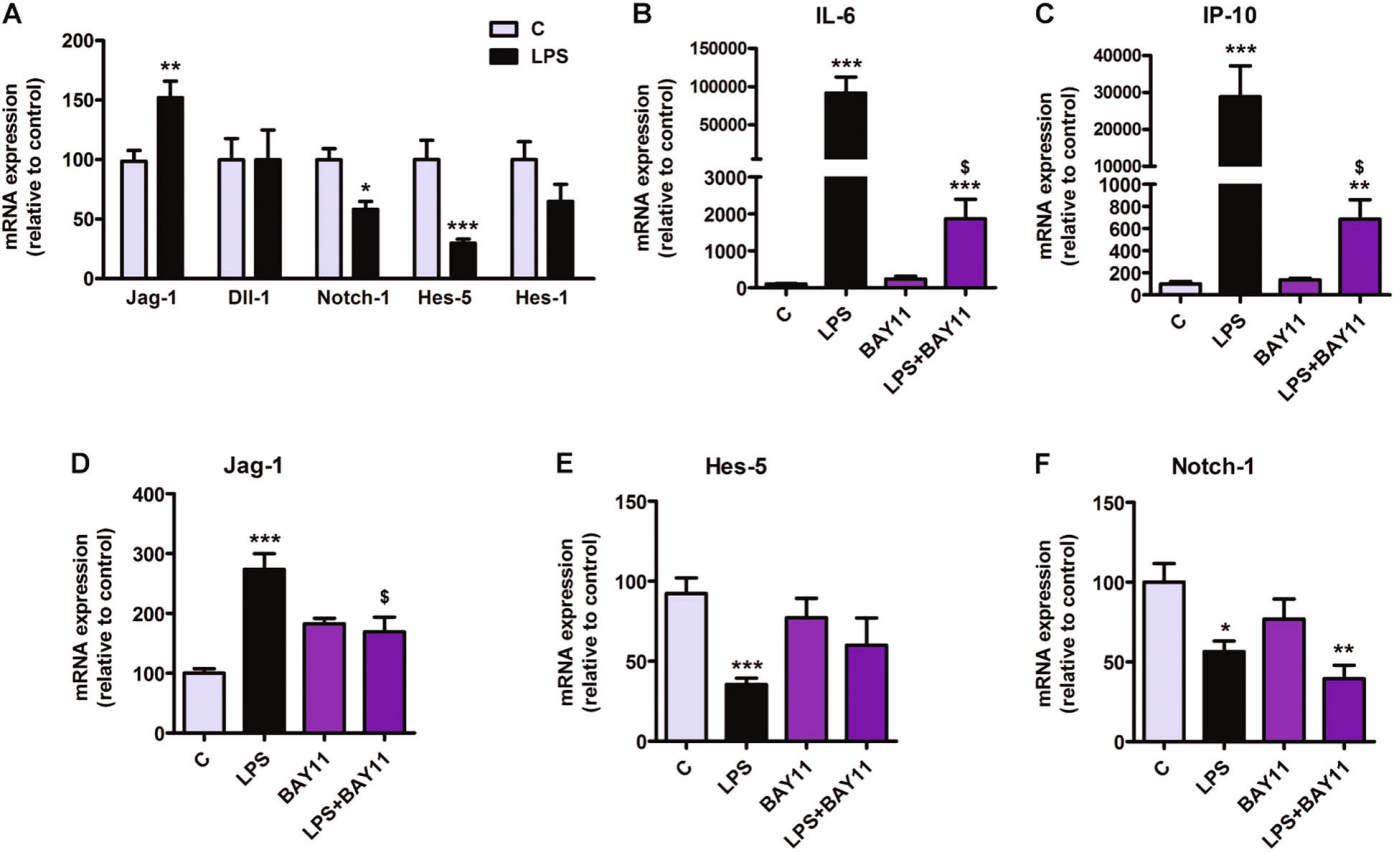
LPS regulates Notch signaling in primary astrocytes. **a** LPS treatment induces the transcription of the ligand Jagged-1, while reduces the expression of the receptor Notch-1 and the effector Hes-5. *, **, *** significant differences (*p* < 0.05, *p* < 0.01, and *p* < 0.001) versus control (Student’s *t-*test, *n* = 4). **b**–**e** The NFκB inhibitor BAY-1 is able to significantly reduce the LPS-induced increase in the transcription of IL-6, IP-10, and Jag-1 (**b**–**d**) and decrease of Hes-5 (**e**). **f** LPS downregulation on Notch-1 transcription is not modified by BAY-11. Statistical significance was determined using one-way ANOVA and Bonferroni post hoc test; *, **, ***significant differences (*p* < 0.05, *p* < 0.01, and *p* < 0.001) versus control. ^$^significant differences (*p* < 0.05) versus LPS

To determine the possible implication of the Notch signaling pathway in the changes induced by LPS in astrocytes, we evaluated the mRNA expression of different genes related with Notch signaling. LPS positively regulated the transcription of the ligand Jagged-1 (Jag-1) while significantly reduced the expression of the Notch-1 receptor and the Notch-1 effector Hes-5 (Fig. 2a). In contrast, LPS did not significantly affect the expression of Dll and Hes-1 (Fig. 2a).

### NFκB activation is involved in the transcriptional regulation of Jag-1 and Hes-5 by LPS

We had already demonstrated that the increase in the expression of pro-inflammatory cytokines by astrocytes in response to LPS is mediated by NFκB activation^30^. Accordingly, in our model, the NFκB inhibitor BAY-11, significantly reduced the effect of LPS on the transcription of IL-6 and IP-10 (Fig. 2b, c). In addition, the upregulation of Jag-1 and the downregulation of Hes-5 induced by LPS were abrogated by this drug (Fig. 2d, e), indicating that the transcriptional effect of LPS on Jag-1 and Hes-5 depends on activation of NFκB. In contrast, the effect of LPS on Notch-1 transcription was not modified by BAY-11 (Fig. 2f).

### Hes-5 downregulation by LPS in astrocytes is mediated by a decrease in NICD

**Fig. 3:**
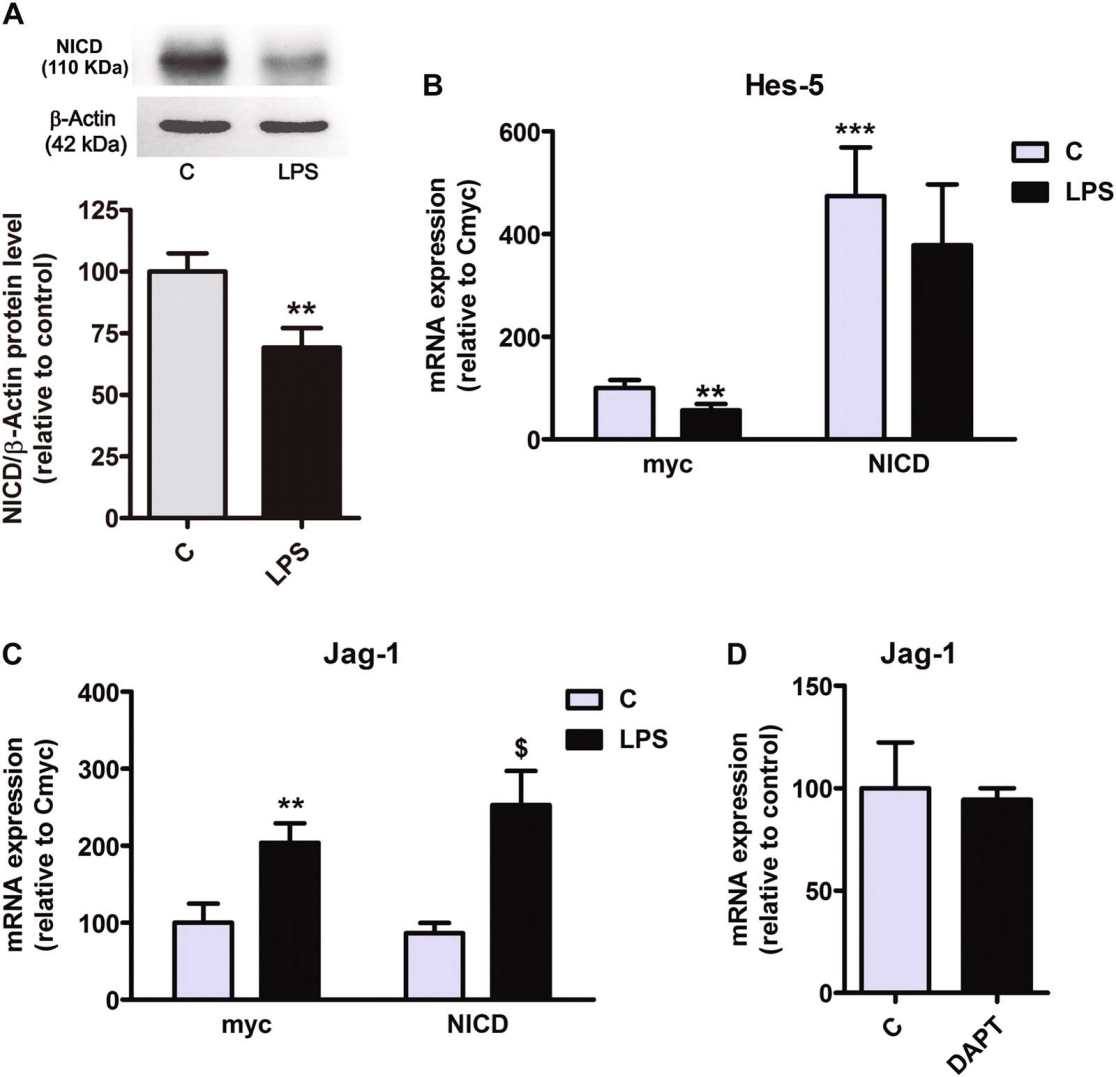
Hes-5 downregulation by LPS in astrocytes is mediated by a decrease in NICD. **a** Representative immunoblot shows NICD expression levels in astrocytes cultures treated with LPS. Actin was used as a loading control. Statistical significance (*p *< 0.01) was determined using Student’s *t*-test, *n* = 7. **b**, **c** The influence of LPS on the expression of Hes-5 and Jag-1 determined by qPCR in astrocytes cultures overexpressing NICD. **, ***Significant differences (*p* < 0.01 and *p* < 0.001) versus control cultures that express myc-tag and ^$^significant difference (*p* < 0.05) versus control that express myc-NICD (Student’s t-test, *n* = 4). **d** Expression of Jag-1 determined by qPCR in cultures treated with DAPT

We also evaluated the levels of the Notch intracellular domain (NICD), which is released when Notch is activated. We observed that the astrocytes stimulated with LPS had lower amounts of NICD than control ones (Fig. 3a). Furthermore, we transfected a NICD-expressing myc-tagged plasmid into primary astrocytes. Constitutive expression of NICD generated an activated Notch phenotype that was confirmed by a significant increase in Hes-5 mRNA expression (Fig. 3b). Interestingly, NICD overexpression prevented the LPS-induced Hes-5 transcriptional downregulation observed in control astrocytes, clearly indicating that Hes-5 downregulation provoked by LPS in astrocytes relies upon Notch-1 processing and NICD release (Fig. 3b).

NICD overexpression in astrocytes did not modify Jag-1 mRNA expression under control conditions, nor prevented its induction by LPS (Fig. 3c). Besides, the addition of DAPT (the γ-secretase inhibitor) to the astrocytic cultures did not alter Jag-1 mRNA expression (Fig. 3d), indicating that Jag-1 transcription is not under the control of Notch-1 receptor activation.

### Notch signaling is involved in the effect of LPS on astrocyte morphology

**Fig. 4:**
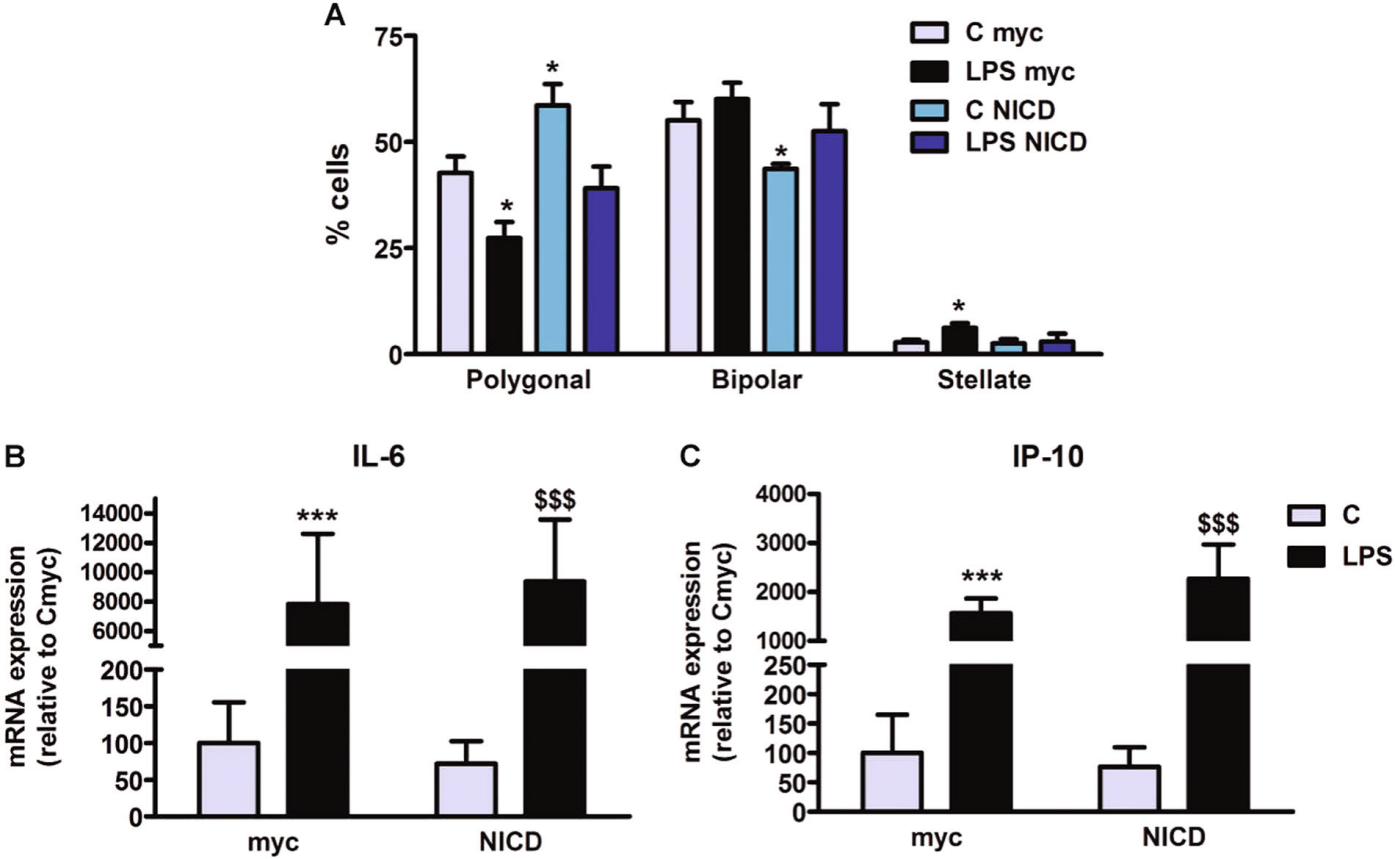
Notch activation counteracts the effect of LPS on astrocytes morphology but not on the transcription of pro-inflammatory cytokines. **a** Notch activation by overexpressing NICD in the culture, changes astrocyte morphology in the opposite way that LPS: increases the proportion of polygonal cells, decreases the proportion of bipolar cells and does not change the stellates. Overexpression of NICD rescues the phenotype to control values in LPS treated astrocytes. Statistical significance was determined using one-way ANOVA and Bonferroni post hoc test for each phenotype separately, *significant differences (*p* < 0.05) versus their respective control, *n* = 4. **b**, **c** Notch activity does not modulate pro-inflammatory cytokines expression and does not interact with the effect of LPS on their expression. ***significant differences (*p* < 0.001) versus control cultures that express myc-tag and ^$$$^significant differences (*p* < 0.001) versus control that express myc-NICD

Astrocytes were also transfected with NICD-expressing plasmid to determine whether Notch signaling is involved in the morphological effects of LPS on these cells. Overexpression of NICD in astrocytes resulted in a significant increase in the proportion of cells with a polygonal morphology and in a significant decrease in the proportion of bipolar cells compared to control astrocytes transfected with the empty vector (Fig. 4a). This suggests that Notch signaling regulates astrocyte morphology under basal conditions. In addition, the treatment with LPS of astrocytes transfected with the empty vector resulted in a significant decrease in the proportion of cells with polygonal morphology and a significant increase in the proportion of cells with stellate morphology compared to control astrocytes (Fig. 4a). The morphological effect of LPS was blocked in the NICD overexpressing astrocytes (Fig. 4a), suggesting that Notch signaling mediates the effect of LPS on astrocyte morphology.

In contrast to astrocyte morphology, the expression of IL-6 and IP-10 under basal conditions and after LPS stimulation was not affected by overexpression of NICD in astrocytes (Fig. 4b, c). This suggests that canonical Notch-1 signaling does not mediate the expression of IL-6 and IP-10 under basal conditions and does not mediate their upregulation by LPS.

### IGF-1 regulates Notch signaling and morphology in astrocytes

IGF-1 is a neuroprotective factor that is known to reduce reactive astrogliosis by the inhibition of NFκB in astrocytes^29,31,32^. Since our previous results indicate that NFκB is involved in the regulation of Notch signaling, we hypothesized that IGF-1 could regulate Notch signaling in astrocytes.

**Fig. 5:**
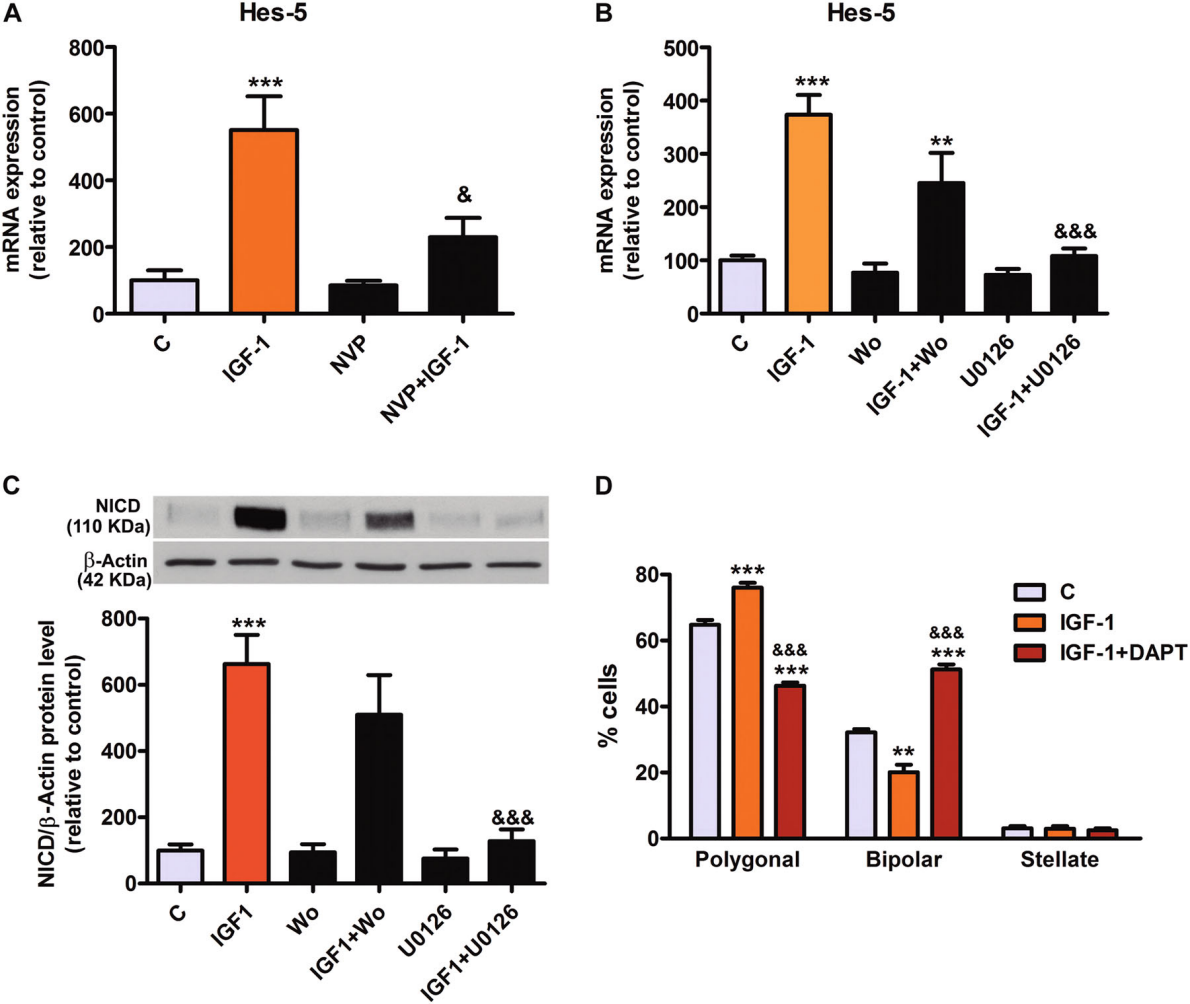
IGF-1 regulates Notch signaling and morphology in astrocytes. **a** IGF-1-induced Hes-5 overexpression involves IGF-1R. **b**. **c** IGF-1 increases Hes-5 transcription (**b**) and NICD production (**c**) through MAPK pathway. **, ***significant differences (*p* < 0.01 and *p* < 0.001) versus control cells. ^&&, &&&^significant differences (*p* < 0.01 and *p* < 0.001) versus IGF-1-treated cells. **d** To determine whether exogenous addition of IGF-1 is able to modify astrocyte morphology, primary cultures were treated with 100 nM IGF-1 alone or in the presence of DAPT. Graph represents the percentage of cell type in each condition. Statistical significance was determined using one-way ANOVA and Bonferroni post hoc test for each phenotype separately, **, ***(*p* < 0.01 and *p* < 0.001) versus their respective control; ^&&&^(p < 0.001) versus IGF-1-treated astrocytes, *n* = 4. Note that the effect of IGF-1 on astrocyte morphology is similar to NICD expression and opposite to LPS treatment

To explore this possibility, astrocyte cultures were treated with the growth factor for 24 h. In addition, some cultures were pre-treated for 4 h with NVP, a specific inhibitor of IGF-1R, and then stimulated with IGF-1 for 24 h. The expression levels of Hes-5 mRNA were measured by real-time PCR. IGF-1 increased the expression of Hes-5 (Fig. 5a) and NVP significantly reduced this effect, indicating that the effect of IGF-1 on the regulation of Notch pathway is mediated, at least in part, by its binding to IGF-1R.

To test whether the PI3K or the MAPK pathways, which are activated by IGF-1R, mediate the effect of IGF-1 on Notch signaling, we stimulated astrocytes in the presence of selective inhibitors of these two signaling pathways. Astrocytes were pre-treated for 4 h with each inhibitor and then stimulated with IGF-1 for 24 h. Levels of Hes-5 mRNA transcription were measured by real-time PCR (Fig. 5b) and the levels of NICD were measured by western Blot (Fig. 5c). IGF-1 significantly increased the levels of Hes-5 mRNA (Fig. 5b) and the levels of NICD (Fig. 5c). Inhibition of the PI3K pathway with wortmannin did not significantly affect the action of IGF-1. However, the blockade of the MAPK pathway with U0126 resulted in a total inhibition of Hes5 expression (Fig. 5b) and NICD production (Fig. 5c).

Since our results indicate that Notch signaling mediates the morphological changes induced by LPS on astrocytes and that IGF-1 regulates Notch signaling, we decided to assess whether IGF-1 regulates astrocyte morphology. Fig. 5d shows that IGF-1 increased the proportion of astrocytes with a polygonal morphology and decreased the proportion of bipolar astrocytes. This effect is opposite to the effect of LPS and seems to depend on Notch signaling since it was blocked by DAPT, indicating that it depends on the γ-secretase activity.

### IGF-1 blocks the effect of LPS on Notch signaling and morphology in astrocytes

**Fig. 6:**
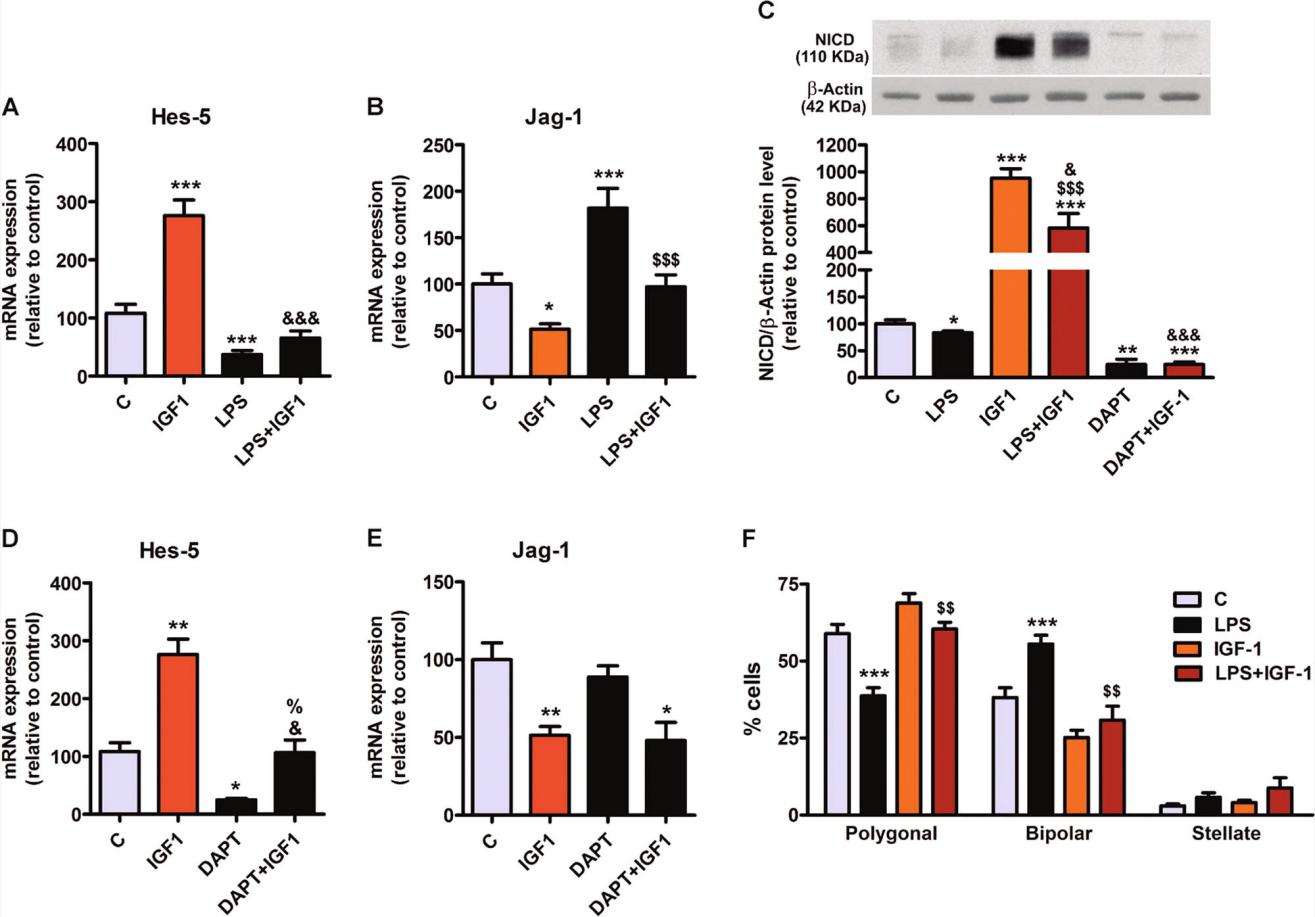
IGF-1 counteracts the effect of LPS on Notch signaling and astrocyte morphology. **a**, **b** Expression levels of Hes-5 and Jag-1 in cortical astrocytes treated with IGF-1 alone or in the presence of LPS. **c**–**e** LPS and gamma-secretase activity mediate IGF-1-induced Noth-1 cleavage (**c**) and Hes-5 (**d**) and Jag-1 (**e**) transcription in cortical astrocytes in vitro. *, **, ***significant differences (*p* < 0.05, *p* < 0.01 and *p* < 0.001) versus control cells; ^&, &&&^significant differences (*p* < 0.05 and *p* < 0.001) versus IGF-1-treated cells; ^$$$^significant difference (*p* < 0.001) versus LPS treated cells and % (p < 0.05) significant difference versus DAPT-treated cells (one-way ANOVA and Bonferroni post hoc test, *n* = 4). **f** IGF-1 is able to counteract the effect of LPS on astrocytes morphology. Graph represents the percentage of cell type in each condition. Statistical significance was determined using one-way ANOVA and Bonferroni post hoc test for each phenotype separately, ***(*p* < 0.001) versus their respective control; ^$$^(*p* < 0.01) versus LPS-treated astrocytes, *n* = 4

Since IGF-1 exerted opposite effects to LPS on Notch signaling, we tested whether IGF-1 could counteract the effect of LPS on Notch signaling in astrocytes. Fig. 6a shows that, in agreement with our previous experiments, the addition of IGF-1 to the astrocyte cultures increased Hes-5 mRNA levels and abrogated the reduced expression of Hes-5 induced by LPS. In addition, IGF-1 significantly decreased the expression of Jag-1 and counteracted the upregulation of Jag-1 by LPS (Fig. 6b).

Astrocytes treated with IGF-1 showed a striking increase in NICD levels (Fig. 6c), indicating a strong Notch activation. LPS was able to significantly reduce NICD levels in the astrocytes treated with IGF-1. However, NICD levels in astrocytes treated with LPS and IGF-1 were much higher than the levels in astrocytes treated with LPS alone. This indicates that Notch maintains a high degree of activation in IGF-1 treated astrocytes even in the presence of LPS. Treatment of astrocytes with DAPT blocked the effect of IGF-1 on NICD levels (Fig. 6c), on Hes-5 expression (Fig. 6d) and on Jag-1 expression (Fig. 6e) suggesting that the effect of IGF-1 on Notch signaling depends on the γ-secretase activity.

As our results indicated that IGF-1 neutralizes the effect of LPS on Notch signaling in astrocytes, we decided to assess whether IGF-1 was also able to block the changes induced by LPS on astrocyte morphology. Fig. 6f shows that IGF-1 counteracted the effect of LPS on the proportion of astrocytes with polygonal and bipolar morphology.

### Jag-1 depletion impairs the effect of LPS on the astrocyte morphology

**Fig. 7:**
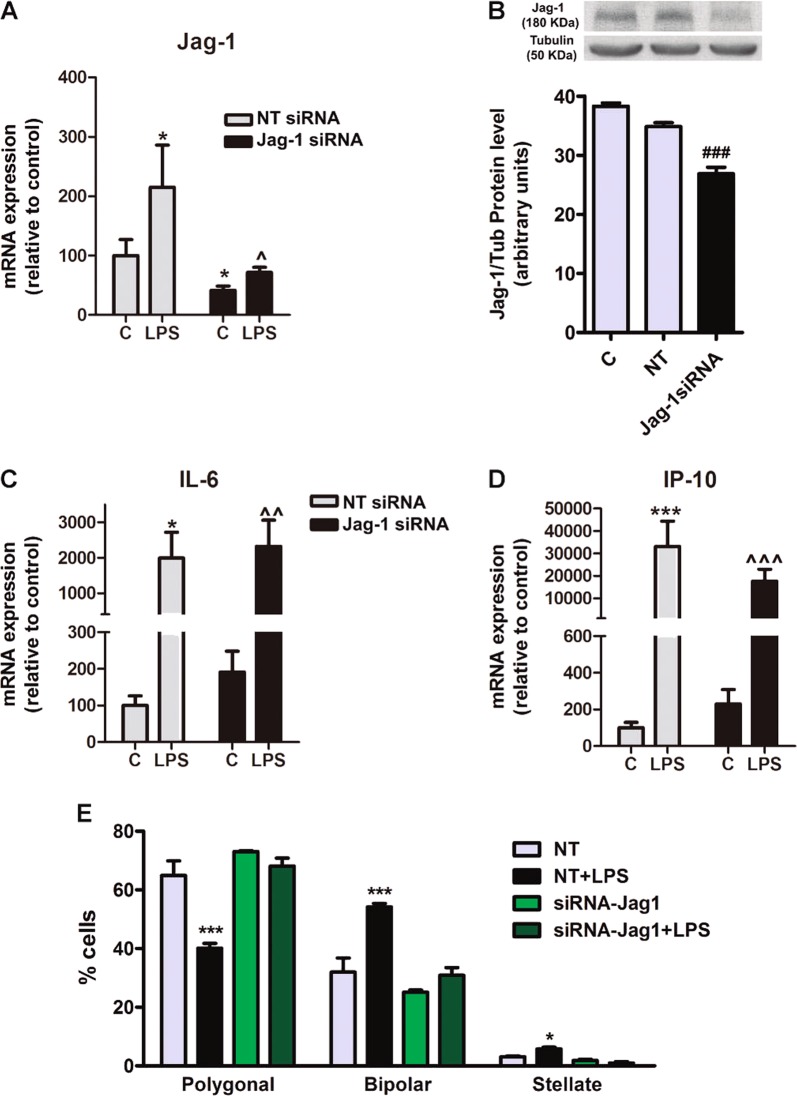
Jag-1 downregulation rescues the effect of LPS on astrocyte morphology but not on the transcription of pro-inflammatory cytokines. Astrocytes were transfected with a specific Jag-1 and a non-target siRNA, and then exposed to LPS. **a**, **b** Jag-1-siRNA significantly decreased both Jag-1 mRNA (**a**) and protein expression (**b**). **c**, **d** Jag-1 downregulation do not alter the expression of pro-inflammatory cytokines under either control or inflammatory conditions. *, ***significant differences (*p* < 0.05 and *p* < 0.001) versus control non-target siRNA; ^, ^^, ^^^^significant difference (*p* < 0.05, *p* < 0.01 and *p* < 0.001) versus control Jag-siRNA and ^###^(*p* < 0.001) significant difference versus non-target siRNA transfected cells (one-way ANOVA and Bonferroni post hoc test, *n* = 4). E. Jag-1 downregulation abolishes the effects of LPS on astrocyte morphology. Statistical significance was determined using one-way ANOVA and Bonferroni post hoc test for each phenotype separately, *, ***(*p* < 0.05 and *p* < 0.001) versus their respective control

The above experiments show that astrocytes treated with LPS have higher levels of Jag-1 expression and more reactive morphology. On the contrary, cultures treated with IGF-1 present Jag-1 downregulation and more resting morphology. To directly assess the role of Jag-1 on astrocyte morphology, we transfected astrocyte cultures with a specific Jag-1-siRNA. The silencing efficacy was confirmed measuring Jag-1 expression by real-time PCR (Fig. 7a) and by western blotting (Fig. 7b). We found that silencing of Jag-1 expression had no effect on IL-6 (Fig. 7c) and IP-10 (Fig. 7d) mRNA levels, under basal conditions and after LPS stimulation. In contrast, Jag-1 silencing impaired the effect of LPS on astrocyte morphology (Fig. 7e).

## Discussion

Our findings indicate that Notch signaling is involved in the reactive morphological changes of astrocytes in response to a pro-inflammatory stimulus, such as LPS. Indeed, our data suggest that the effects of LPS on astrocyte morphology depend on the upregulation of Jag-1 by NFκB. In turn, Jag-1 upregulation causes the downregulation of NICD, which mediates the change in astrocyte morphology. In contrast, Notch signaling is not involved in the upregulation of the pro-inflammatory molecules IL-6 and IP-10 in response to LPS.

Our results show that LPS decreases the expression of Hes-5 and Notch-1 and increases the expression of Jag-1, but does not affect the expression of Dll. The increased expression of Jag-1 by LPS is in concordance with previous results that shown that Jag-1 is under the control of the NFκB signaling pathway^33,34^ and that LPS activates NFκB in astrocytes by promoting the translocation of p65/NFκB subunit to the cell nucleus^30^.

Canonical Notch signaling is initiated by ligand binding to the Notch receptor on neighboring cells, which leads to the proteolytic processing of the receptor and the release of NICD. Nevertheless, our experiments demonstrate that LPS induces changes in astrocyte morphology by blocking Notch signaling, while enhancing the mRNA expression of Jag-1. Jagged ligands have been described as inhibitory or antagonistic for the activation of Notch signaling by Dll^35^. Several studies demonstrated that Dll and Jagged may have opposite functions. In angiogenesis, activation of Notch by Dll4 inhibits tip cell selection^36^. In contrast, Jagged-Notch signaling promotes tip cell selection and sprouting by antagonizing Dll4-Notch signaling^35,37,38^. These findings clearly show that the balance between the two Notch ligands is a key factor in cell fate definition and in cell morphology acquisition. Inhibition of Notch signaling by γ-secretase inhibitors or Dll blockade produces hyperbranching in endothelial cells; however, Jagged inhibition decreases branching and reduces angiogenesis^37,39^. The attenuation of Notch signaling induced by Jag-1 can be attributed to cis-inhibition by Jag-1 binding to the Notch receptor and inhibition of the signal mediated Dll in receiver cell^40–43^.

Considering the different function of the two Notch ligands, the LPS-induced altered balance in the expression of Jag-1 and Dll in astrocytes may explain that the increase in Jag-1 expression after LPS is associated with lower levels of NICD and Hes-5, indicating an inhibition of Notch signaling. When NICD was overexpressed in astrocytes, LPS was unable to modify the expression of Hes-5, indicating that the effect of LPS on Notch activity is produced by the canonical signaling pathway^15^. Furthermore, Jag-1 silencing or NICD overexpression in astrocytes resulted in a resting cellular phenotype after LPS stimulation, suggesting that increased Jag-1 expression and the consequent reduction in Notch activity mediates the effect of LPS in the induction of reactive morphology.

The involvement of Notch signaling in the morphology of astrocytes after LPS stimulation may be relevant for the mechanisms of reactive gliosis. Thus, reactive astrocytes present hypertrophy of cell body and increase the thickness of their main cellular processes^5,44^. Further studies should determine whether Notch signaling is also involved in the changes in morphology of microglia, which upon stimulation changes from a ramified, quiescent morphology to an amoeboid, activated morphology^45^.

**Fig. 8:**
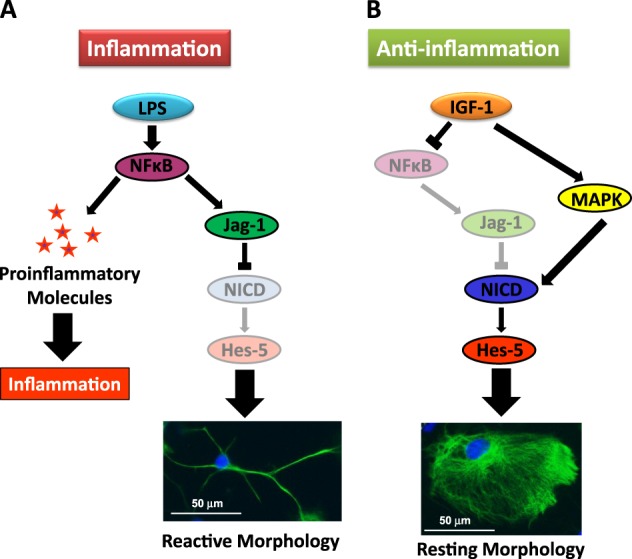
Summary of the effects of LPS and IGF-1 on Notch signaling in astrocytes. **a** Inflammatory conditions in primary astrocytes: LPS activates NFκB that promotes the expression of (1) molecules that generate inflammation and (2) the Notch ligand Jag-1 that reduces Notch activity and consequently induce a reactive morphology in astrocytes. **b** Anti-inflammatory conditions in primary astrocytes: IGF-1 produces an increase of Notch activity by: (1) activating MAPK signaling pathway and (2) reducing Jag-1 NFκB-dependent expression. Both IGF-1 effects drive to enhance Hes-5 expression and result in a resting morphology in astrocytes

In microglia cells, Notch signaling amplifies the pro-inflammatory response by enhancing NFκB/p65 signaling^23,24^, suggesting that both pathways synergistically regulate the inflammatory function in activated microglia. In contrast, in astrocytes our findings indicate that this signaling pathway is not involved in the effect of LPS on the expression of pro-inflammatory molecules. This further suggests that the regulation of Notch signaling in astrocytes by LPS is downstream of NFκB activation since the inhibition of NFκB with BAY-11 blocked the upregulation of Jag-1 and the downregulation of Hes-5 by LPS. Previous studies have shown that LPS-mediated production of cytokines by astrocytes involves the activation of the NFκB pathway^30,46,47^. Thus, we may hypothesize that NFκB stimulates neuroinflammation in reactive astrocytes by the upregulation of pro-inflammatory molecules and, in parallel, by the upregulation of Jag-1, which induces reactive morphological changes in astrocytes (Fig. 8a).

IGF-1 regulates inflammation in a context-dependent manner. In the brain, several studies showed anti-inflammatory effects of IGF-1 acting on astrocytes and microglia and revealed that IGF-1 levels may be critical for regulating the neuroinflammatory response^48^. Experiments in vivo show that the level of IGF-1 increases at the injury site, but at least part of it comes from the periphery in response to brain damage^49^. Following this paradigm, we analyzed the effect of exogenous IGF-1 in astrocytes. Our findings indicate that Notch signaling in astrocytes also mediates their morphological transformation induced by IGF-1. In contrast to LPS, IGF-1 induced a significant decrease in the expression of Jag-1 and a significant increase in the levels of NICD and the expression of Hes-5. Thus, IGF-1 and LPS had opposite effects on Notch signaling in astrocytes (activation and inhibition, respectively). In agreement with this, IGF-1 and LPS exerted also opposite effects on astrocyte morphology. Thus, IGF-1 decreased, while LPS increased, the proportion of astrocytes with reactive morphology, an effect that was mediated by γ-secretase. In addition, IGF-1 was able to counteract the effect of LPS on Notch signaling and on astrocyte morphology. Since Jag-1 expression is regulated by NFκB signaling (our present findings) and NFκB pathway is blocked by IGF-1^31^, we may hypothesize that the action of IGF-1 on Notch signaling is upstream of NFκB. Nevertheless, the IGF-1 induced decrease in Jag-1 expression is not enough to explain the huge accumulation of NICD generation and the change in astrocyte morphology. Jag-1 mRNA downregulation in astrocytes was achieved by siRNA and by IGF-1 treatment, however, only the later was able to increase the percentage of cells displaying less reactive shapes. Blockade of MAPK pathway completely suppress IGF-1 production of NICD as well as increased expression of Hes-5. So our results suggest that actions of IGF-1 on astrocytes involve both, blockade of NFκB pathway and activation of MAPK signaling (Fig. 8b).

In summary, our findings demonstrate for the first time, that Notch signaling is involved in the morphological changes induced in astrocytes by inflammatory and anti-inflammatory factors, expanding the known roles of Notch to the regulation of astrocyte morphology.

## Materials and methods

### Animals

Postnatal day 0 (PND0)–PND2 male and female CD1 mouse pups were raised in our in-house colony at the Cajal Institute. Male pups were distinguished from female pups by a larger genital papilla and longer anogenital distance. All the procedures applied to the animals used in this study were in accordance with the European Commission guidelines (2010/63/UE) and the Spanish regulation (R. D. 53/2013) on the protection of animals for experimental use. These procedures were approved by our institutional animal care and use committee (Comité de Ética de Experimentación Animal del Instituto Cajal) and the Consejería del Medio Ambiente y Territorio (Comunidad de Madrid, PROEX 200/14).

### Cortical astrocyte cultures

Astrocytes were cultured from male and female PND0–PND2 pups (50% each). The brain was extracted, meninges were removed, and the cerebral cortex was isolated under a dissecting microscope and then mechanically dissociated and washed twice in Hank’s balanced salt solution (Sigma-Aldrich, Tres Cantos, Madrid). After complete dissociation in Dulbecco’s modified Eagle’s medium/Nutrient mixture F-12 (DMEM/F-12) culture medium with phenol red (Sigma-Aldrich) containing 10% fetal bovine serum (FBS, Invitrogen, Carlsbad, CA) and 1% antibiotic–antimycotic (Invitrogen), the cells were filtered through a 40 μm nylon cell strainer (Corning Inc., Corning, NY). The cells were centrifuged, resuspended in the same medium, and plated onto poly-L-lysine-coated 75-cm^2^ flasks at 37 °C and 5% CO_2_. The medium was replaced after the first day in vitro and twice per week until the cells reached confluence (~7 days). Then, the cell cultures were shaken overnight at 37 °C and 280 rpm on a tabletop shaker (Infors HT, Bottmingen, Switzerland) in order to minimize oligodendrocyte and microglia contamination. The astrocytes were incubated with 0.5% trypsin (Sigma-Aldrich), centrifuged, resuspended in DMEM/F-12 with 10% FBS and 1% antibiotic–antimycotic, and seeded in poly-L-lysine-coated 75-cm2 flasks at 37 °C and 5% CO2. When the cells reached confluence for the second time (~ after 5 days), the subculture process was repeated but the astrocytes were plated onto poly-L-lysine-coated plates (6, 24, or 48 wells) or glass coverslips using DMEM/F-12 with 10% FBS and 1% antibiotic–antimycotic. Using this protocol we obtained cultures with less than 4% of Iba-1 positive cells, checked by double immunocytochemistry with anti-Iba I (microglial marker) and anti-GFAP antibodies (astroglial marker). Fibroblast contamination was also assessed by immunocytochemistry using an anti-Thy 1 antibody (against CD90, 1:500), but no staining was detected.

### Cell treatments

When cells were confluent in the multiwell plates, they were rinsed once with pre-warmed PBS and then the treatments were applied in DMEM-F12 without additives. Cells were pre-treated with the inhibitory drugs (Table 1) for 4 h, and then IGF-1 (100 nM), and/or LPS (500 ng/mL) were added to the culture medium for 24 h.

**Table 1:** Inhibitory drugs

Target	Inhibitor	Concentration	Supplier
IGF-1R	NVP	400 nM	Cayman Chemical
PI3K	Wortmannin	100 nM	Calbiochem
MEK 1/2	U1026	10 µM	Cell Signaling
γ-secretase	DAPT	2,5 µM	Calbiochem
NFκB	BAY-11	10 µM	Calbiochem

### Cell viability assay

The non-cytotoxic dose of LPS used for all the experiments was set after the analyses of a dose-viability curve using LPS at final concentrations from 50 to 5000 ng/mL in the culture. To assess cell viability, we performed the fluorescein diacetate (FDA)/propidium iodide (PI) assay. Cells were seeded in 24-well plates and treated for 24 h with increasing concentrations of LPS (in phenol red-free DMEM-F12 medium without additives). Just before the end of the treatment, cells were incubated for 50 min at 37 °C with FDA (100 μM) and PI (15 μM). Fluorescence at 520 and 620 nm wavelength was measured in a plate reader (FluoStar OPTIMA, BMG Labtech, Germany).

To evaluate the cells metabolic activity (mitochondrial respiration), the 3-(4,5-dimethylthiazol-2-yl)-5-(3-carboxymethoxyphenyl)-2-(4-sulfophenyl)-2H-tetrazolium, inner salt (MTS) assay was performed with the same LPS concentrations (50–5000 ng/mL). Astrocytes were plated in 48-well microplates and treated for 24 h with LPS at concentrations of 50, 100, 500, 1000, or 5000 μg/mL. After the addition of 20 μL of CellTiter 96 AQueous One Solution (Promega, Madison, USA), the plates were incubated for 4 h at 37 °C and 5 % CO_2_. Absorbance at 490 nm wavelength was measured in a plate reader.

### Proliferation assay

Astrocytes were seeded in glass coverslips (pre-coated with poly-l-lysine) at a density of 25,000 cells/cm^2^ and exposed to a 30 min-pulse of BrdU (bromodeoxyuridine, 10 µM) in order to assess cell proliferation. BrdU incorporation was detected by double immunocytochemistry using antibodies against BrdU and GFAP. Briefly, paraformaldehyde-fixed cells were incubated with 2 N HCl/ 0.5% triton X-100 during 30 min at room temperature. Then, pH was neutralized with sodium tetraborate and primary and secondary antibodies were added.

### Transfections

Astrocytes were transfected at 60% of confluence using the Effectene Transfection Reagent (Qiagen GmbH, Hilden, Germany), following the manufacturer’s instructions. Cells were transfected with a pcDNA1 vector encoding a myc epitope and NICD^50^ using the empty vector as control; other cultures were co-transfected with pmax-GFP plus small interfering RNA (siRNA) oligonucleotide targeted to Jag-1. After 20 h of expression time, cells were treated with the indicated drugs for each assay during 24 h. Then the astrocytes were harvested and processed for real-time polymerase chain reaction (PCR) and western blot analysis or fixed in 4% paraformaldehyde in 0.1 M phosphate buffer for immunostaining.

SiRNA oligonucleotides were purchased from Applied Biosystems/Ambion and the concentration was 30 nM during transfection. SiRNAs targeting Jag-1 was Silencer® Select siRNA ID # s68530. A non-targeting siRNA was used as negative control (Silencer® Select Negative Control #1 siRNA, catalog number 4390843).

### Quantitative RT-PCR

Total RNA was extracted from cultures with Illustra RNAspin Mini RNA isolation kit from GE Healthcare (Buckinghamshire, UK). First strand cDNA was prepared from RNA using the Moloney murine leukemia virus reverse transcriptase (Promega Corp., Madison, Wisconsin) following the manufacturer’s instructions. Quantitative PCR reactions were carried out on an ABI Prism 7500 Sequence Detector (Applied Biosystems, Weiterstadt, Germany) using the TaqMan or Sybr Green Universal PCR Master Mix. TaqMan probe and primers for Hes-5 were Assay-on-Demand gene expression products (Applied Biosystems). Primer sequences for the rest of genes evaluated and control housekeeping gene 18S rRNA, were designed using Primer Express (Applied Biosystems) (Table 2). All reactions were done in duplicates, from at least 4 different cultures. Gene expression was normalized for 18S rRNA expression. The ΔΔCT method was used for relative quantification analysis.

**Table 2:** Primer sequences for real-time polymerase chain reaction

Gene Symbol	Forward 5´–3´	Reverse 5´–3´
18S-rRNA	CGCCGCTAGAGGTGAAATTCT	CATTCTTGGCAAATGCTTTCG
Hes-1	CCAGCCAGTGTCAACACGA	AATGCCGGGAGCTATCTTTCT
Notch-1	CCCTTGCTCTGCCTAACGC	GGAGTCCTGGCATCGTTGG
Dlk-1	AATGTCTGCAGGTGCCATGTT	TGCACTGCCATGGTTCCTT
Jag-1	TCAGGACACACAACTCGGAAGT	CTCCTCTCTGTCTACCAGCGTATACA
IGF-1	GTGATCTGAGGAGACTGGAGATGTACT	TGAGTCTTGGGCATGTCAGTGT
IL-6	GAAACCGCTATGAAGTTCCTCTCTG	TGTTGGGAGTGGTATCCTCTGTGA
IP-10	CAGGAGAATGAGGGCCATAGG	CGGATTCAGACATCTCTGCTCAT

### Western blot

Primary cultures were lysed in 150 µL of Laemmli buffer, heated during 5 min at 100 °C and sonicated for 5 min. Solubilized proteins (30 µL) were resolved in 8–10% SDS-PAGE bis-acrylamide gels and transferred to nitrocellulose membranes (Trans-Blot turbo transfer pack, Biorad) in a semi-dry system (Trans-Blot Turbo Transfer System, Biorad). Membranes were blocked for 2 h in a 5% BSA-TTBS (138 mM NaCl, 25 mM Tris-HCl, pH 8.0 and 0.1% Tween-20) solution, and incubated overnight with the primary antibodies (Table 3) at 4 °C under moderate shaking. Anti-β-actin, anti-Tubulin and anti-GAPDH mouse monoclonal antibodies were used as loading controls. All secondary antibodies were from Jackson Immuno Research (West Grove, PA, USA). Proteins were visualized with a chemiluminescence detection reagent according to the manufacturer’s instructions (Amersham, GE Healthcare Europe, Barcelona, Spain). The densitometric analysis of scanned films was performed with ImageJ software (Maryland, USA. http://imagej.nih.gov/).

**Table 3:** Antibodies used

Antigen	Host	Dilution	Source
NICD, cleaved at Val 1744	Rabbit	1:500	Cell Signaling
Jagged-1	Rabbit	1:500	Santa Cruz
β-actin	Mouse	1:5000	Sigma-Aldrich
α-Tubulin	Mouse	1:5000	Sigma-Aldrich
Iba1	Rabbit	1:2000	Wako
GFAP(GA5 clon)	Mouse	1:500	Sigma-Aldrich
BrdU	Mouse	1:50	Hybridoma Bank
GFAP	Rabbit	1:500	Dako
c-myc (9E10)	Mouse	1:500	Roche
Thy-1 (against CD90)	Mouse	1:500	Bio-Rad (Formerly AbD Serotec)
Anti-mouse-HRP	Goat	1:10000	Jackson Laboratories
Anti-rabbit-HRP	Goat	1:10000	Jackson Laboratories
Anti-mouse Alexa 488	Goat	1:1000	Jackson Laboratories
Anti-rabbit Alexa 594	Goat	1:1000	Jackson Laboratories
Anti-mouse Alexa 647	Goat	1:50	Invitrogen

### Immunocytochemistry

Cells were seeded in glass coverslips (pre-coated with poly-l-lysine) at a density of 25,000 cells/cm^2^. After the appropriate treatments, astrocytes were fixed for 20 min with 4% paraformaldehyde at room temperature and permeabilized for 4 min with 0.12% Triton-X plus 0.12% gelatin in PBS. Cells were then washed with PBS/gelatin and incubated for 1 h with primary antibodies (Table 3). After washing in the same buffer, cells were incubated for 45 min at room temperature with the proper fluorescent secondary antibodies (Table 3). For morphology assessment, GFAP-positive cells were classified into three different categories: Polygonal astrocytes were those without any cytoplasmic protrusions, Bipolar astrocytes presented an elongated cell body or one thin and long protrusion, and Stellate astrocytes were those with a reduced cell body and three or more long ramifications (Fig. 1e).

### Statistical analysis

Data shown in the figures are the result of 4–10 independent experiments and are presented as the mean ± standard error of the mean (SEM). Statistical analyses were carried out using SPSS Statistics 23 software (IBM, Armonk, NY). Gaussian distribution of data sets was assessed by Kolmogorov–Smirnov test. Statistical significance was evaluated by the unpaired Student´s *T*-test for one to one comparisons, and by two-way analysis of variance (ANOVA) followed by Bonferroni or Games-Howell post hoc tests (depending on whether variances were homogeneous or not, respectively) for multiple comparisons. When an interaction between two factors was not detected, data were split and each factor was analyzed by one-way ANOVA followed by Bonferroni or Student’s *t*-test. The statistical significance level was set at *p* < 0.05.
